# An exploration of relationships between associative and non-associative measures of inhibition

**DOI:** 10.1177/17470218241310859

**Published:** 2025-01-13

**Authors:** Ovidiu I Brudan, Hedwig Eisenbarth, Steven Glautier

**Affiliations:** 1 University of Southampton, Southampton, UK; 2 Victoria University of Wellington, Wellington, New Zealand

**Keywords:** associative learning, conditioned inhibition, occasion setting, inhibition, feature negative discrimination

## Abstract

Conditioned inhibition and occasion setting are two examples of inhibitory associative phenomena that have traditionally been studied in isolation from non-associative inhibition. Non-associative inhibition has been assessed using a variety of measures (e.g., stop signal reaction time and impulsivity questionnaires) and weak non-associative inhibition has been linked to a variety of disorders including addiction. However, even though both associative and non-associative inhibition have a common core—both involve suppression of behaviour, there has been relatively little study of potential relationships between these different forms of inhibition. In the current investigation, we carried out exploratory analyses to look for possible links between associative inhibition and four non-associative measures of inhibition, namely, (1) stop signal reaction time, (2) delay discounting, and scores on (3) the Behaviour Inhibition System/Behaviour Activation System and (4) Barratt Impulsivity questionnaires. Despite the fact that we carefully selected data to minimise noise in the measurement of associative inhibition, we found no clear evidence of links between associative and non-associative inhibition. We therefore conclude that while there may be superficial similarities between these different forms of inhibition they are likely to have different substrates.

Associative learning processes allow organisms to adapt to changes in the environment, and inhibitory associative learning is one way to conditionally modify previously learnt behaviours (see Sosa (2022) and Williams (1995) for reviews of inhibitory associative learning phenomena). Conditioned inhibition and negative occasion-setting are forms of associative inhibition that can be established when an organism learns that a specific stimulus signals the omission of an otherwise expected event as seen in a simple feature-negative (FN) procedure as follows. Take the example of a rat which learns that it will receive food every time a light flash occurs (A+ trials). In traditional Pavlovian terminology the light is a conditioned stimulus and the food is an unconditioned stimulus, alternatively known as cue and outcome, respectively. If, on some trials, cue A is presented together (in compound) with a second cue, B, a tone, and the outcome does not occur (AB− trials) then cue B may become a conditioned inhibitor or an occasion-setter (Bouton, 1997; Holland, 1992; Rescorla, 1987). As a result, the rat will no longer respond as if it was expecting food on the AB trials. The main difference between B as a conditioned inhibitor and B as an occasion-setter is that the response inhibiting properties of a conditioned inhibitor are general so that responding to a CB compound (a summation test), after C+ trials, would also be suppressed. If B’s inhibitory properties were specific to A, then B would be said to have acquired occasion-setting properties (Holland, 1992).

Whether or not training in an FN discrimination will result in the feature (cue B in this example) acquiring conditioned inhibition or occasion setting properties can be determined by procedural as well as individual difference variables. In the case of procedural variables, serial presentation of cues (B then A) is more likely to lead to cue B becoming a specific negative occasion setter for cue A than simultaneous presentation of A and B. In contrast, simultaneous presentation tends to result in B becoming a general conditioned inhibitor (Holland, 1992; Swartzentruber, 1995). Recent studies with human participants have provided evidence that there are individual differences in “strategy” adopted given fixed procedures (Glautier & Brudan, 2019; Lee & Lovibond, 2021). To expand, in Experiment 1 of the study by Glautier and Brudan, participants were classified as inhibitors or non-inhibitors based on a summation test carried out in a context that had been used for extinction. In Experiment 2, FN performance of those who had been classed as inhibitors in Experiment 1 was disrupted more than the performance of the non-inhibitors by reinforcing the feature. This pattern would be expected if the inhibitors and non-inhibitors had learned conditioned inhibition and occasion-setting, respectively, because reduced responding to the target by the presence of the feature relies on the feature’s association with the outcome in the case of conditioned inhibition. In contrast, for occasion setting, the feature does not control responding by its association with the outcome. Instead, the feature appears to control the operation of the target-outcome association (c.f. Bonardi et al., 2017; Bouton, 1994; Nelson, 2002, for further analysis).

Inhibitory phenomena are not unique to the domain of associative learning. For example, in the literature on impulsivity there is frequent reference to behavioural inhibition which in various forms incorporates a wide range of phenomena including those that fall under the headings of impulsive actions and impulsive choices (Bari & Robbins, 2013). Elaborating further, inhibitory processes in the context of impulsive action would facilitate stopping responses that have already been initiated, and in the context of impulsive choice would facilitate waiting for delayed rewards (e.g., Bari & Robbins, 2013; Broos et al., 2012).

In this article, we were interested in connecting these two areas of research by exploring the relationship between associative inhibition produced in an FN predictive learning task as traditionally studied in relation to associative learning under the headings of occasion-setting and conditioned inhibition, and inhibition as traditionally studied in other domains. Surprisingly, as noted by Sosa and dos Santos (2019; c.f. also Sosa, 2022), there are few studies that have assessed the relationships between the aforementioned forms of inhibition, and in the studies that have, the results have been mixed (He et al., 2011, 2013; Migo et al., 2006).

Migo et al. (2006) assessed the relationship between conditioned inhibition and scores on Carver and White’s (1994) Behaviour Inhibition System/ Behaviour Activation System (BIS/BAS) scales. They unexpectedly found that conditioned inhibition was positively correlated with the BAS-reward responsiveness subscale but no relationship was found between conditioned inhibition and BIS (nor with the other BAS subscales). He et al. (2011) also assessed the relationship between conditioned inhibition and other forms of inhibition by comparing a group of individuals with a history of offending who were characterised by impulsive/violent behaviour to a control group from the general population using their performance on a conditioned inhibition task. The control group showed a conditioned inhibition effect while the group with a history of offending did not, suggesting that weak conditioned inhibition may be linked to impulsive behaviour. In a follow-up study He et al. (2013) examined the relationship between conditioned inhibition and the BIS/BAS scales in a sample of university students. They found no relationship between inhibitory learning and BAS, failing to replicate the unexpected result noted above, but they did report a significant negative correlation between the BIS and inhibitory learning. This result was, once again, unexpected based on the assumption that there is a common process underlying conditioned inhibition and response inhibition as measured with the BIS subscales. Thus, as shown in these examples the relationship between conditioned inhibition and the BIS/BAS is not as clear as it might be, but there is some evidence of weaker conditioned inhibition in offenders with a history of impulsive behaviour.

Therefore, our goal in the current investigation was to assess further the evidence for a common inhibitory process that contributes to performance across different domains of inhibition. In particular, we focussed on the relationship between associative inhibition acquired in an FN learning task and four “non-associative” measures of inhibition: (1) stopping responses that have already been initiated using the Stop-Signal Reaction Time task (SSRT) and (2) stopping responses that would lead to the choice of smaller-sooner rewards to obtain larger rewards in a delay-discounting task. These were selected as examples of non-associative measures of inhibition because of their currency in the literature and, in the case of the SSRT, because the task itself closely resembles the procedure used in FN learning tasks. We also looked at the relationship between associative forms of inhibition and two widely used questionnaire-based measures (3) the Behavioural Inhibition System/Behavioural Activation System (BIS/BAS) questionnaire (Carver & White, 1994; Patterson & Newman, 1993) and (4) the Barratt Impulsivity Questionnaire (BIS-11; Patton et al., 1995). The BIS/BAS questionnaire is derived from Gray’s (1982) reward sensitivity theory, which involves the interaction of a behavioural inhibition system and a behavioural activation system. The BIS is assumed to react to novel stimuli and signals for non-reward and punishment by inhibiting ongoing behaviour and this is reflected in the BIS subscales of the BIS/BAS questionnaire. The BIS subscales have items to assess sensitivity to stimuli which are anxiety and fear provoking (Carver & White, 1994; Gray, 1987). The BAS is assumed to react to reward, non-punishment and punishment avoidance by activating reward-related behaviours. Correspondingly, the BAS subscales of the BIS/BAS have items which assess sensitivity to reward-related stimuli (Carver & White, 1994). The BIS-11 assesses impulsivity on a number of sub-scales (e.g., motor, self-control) which contain items directly relevant to inhibition as a complement of impulsivity (e.g., “I act on impulse,” “I am self-controlled”). We use the term “associative inhibition” as a way to articulate the distinction between, in particular, the forms of inhibition commonly studied in Pavlovian preparations and “the rest.” Of course associative processes are involved in the SSRT and in delay discounting (and indeed in practically every meaningful behavioural process) but predictive Pavlovian tasks are optimal for identifying the important associative structures that have been proposed to underlie associative inhibition, namely, conditioned inhibition and occasion setting, wherein as noted above, a conditioned inhibitor works via an inhibitory associative connection with the outcome representation whereas an occasion-setter works via an inhibitory associative link that controls and association between a cue and an outcome representation.

To separately assess conditioned inhibition and occasion setting our procedures for evaluating associative inhibition involved two stages. First, we assessed associative inhibition defined by performance in FN discriminations. However, as previously mentioned, solving FN discriminations could be due to the participant learning conditioned inhibition or occasion setting but these possibilities cannot be distinguished purely based on the FN discrimination performance. Therefore, in the second stage, conditioned inhibition was assessed in summation tests. These summation tests gave us a direct measure of the extent to which each participant had developed conditioned inhibition during the FN phase but, in addition, enabled us to classify participants as inhibitors and non-inhibitors. Since inhibitory and non-inhibitory strategies are relatively stable within individuals (Glautier & Brudan, 2019) we were then able to return to the FN discrimination and examine separately the performance of inhibitors and non-inhibitors. We use the terms “inhibitors” and “non-inhibitors” in the foregoing to distinguish those participants who passed and failed our summation test, respectively, after acquiring the FN discrimination and we consider it likely that inhibitors had developed conditioned inhibition during the FN discrimination and that the non-inhibitors had solved the FN discrimination using occasion-setting strategies. However, although the specificity of a cue’s inhibitory properties is a primary marker of occasion-setting other tests could be applied to strengthen this conclusion. For example, a negative occasion-setter should not lose its occasion-setting properties if it is paired with the outcome (Glautier & Brudan, 2019; Holland, 1992). Since we only applied the specificity criterion the term “non-inhibitor” to avoid full commitment to categorising participants as occasion-setters while preserving an important individual differences distinction.

## Method

### Participants

Due to the fact that we did not know at the outset how many participants would meet our inclusion criteria (see “Data selection and analysis” section), we could not carry out accurate a priori power analysis to determine sample size. However, an initial exploratory power analysis indicated 122 participants would be needed to detect a medium effect size for a multiple regression model with seven predictors with power > 80%. Recruitment was carried out until this number was exceeded and funding was secured to allow for a 10% exclusion rate yielding an initial sample of 133 participants (of which 70 identified as male, 60 identified as female, and 3 preferred not to say, the mean age was 35 years, *SD* = 13) recruited via Prolific (https://www.prolific.co/). They were each paid £2.50 for taking part in an online experiment that involved completing a series of questionnaires and behavioural tasks which, altogether, took approximately 30 min to complete. All tasks were presented on web servers running at the University of Southampton.

### Questionnaires

Three questionnaire-based measures were used: (1) The BIS/BAS scales (Carver & White, 1994), (2) the BIS-11 (Patton et al., 1995), and (3) an adjusting amount delay discounting questionnaire. The delay discounting questionnaire consisted of 10 blocks of choices between hypothetical monetary rewards. Each choice was between a smaller immediate reward and a later larger reward. The blocks used five delays: 1 week, 1 month, 6 months, 1 year, and 2 years. Each delay was used twice, once in an ascending block and once in a descending block. Questions were all of the form “Would you prefer S now or L in D?” where S was a (variable) small sooner reward value, L was a (fixed) large later reward value and D was the delay until L. In ascending blocks S started at £5 and each time the participant chose L, S would increase in the next question until chosen. S was one of £5, £100, £250, £550, £800, £950, £990, and £1,000 whereas L was always £1,000. In descending blocks S started at £1,000 and each time the participant chose S it would be decreased in the next question until L was chosen. This procedure allowed the estimation of indifference points (average of indifference points obtained in ascending and descending sequences) at each delay, following which, least-squares non-linear regression was used to fit Mazur’s hyperbolic delay discounting (Equation 1) to the indifference points (Mazur, 1987). From Equation 1, we obtained a discounting parameter *k* for each participant, which was used as a measure of response inhibition—larger *k* values suggest weak response inhibition, corresponding to a pattern of impulsive choices biased towards smaller sooner rewards. In Equation 1, V is the estimated value of the larger later reward (the indifference point) at delay D given L the “now” value of the larger later reward (£1,000) and *k* is the estimated discounting parameter

(1) $$V=\frac{L}{1+kD}$$

### Stop signal reaction time task

An online version of the STOP-IT task (Verbruggen & Logan, 2008) was used to measure SSRT as an index of response inhibition capacity. The task was developed following principles highlighted in a guide for measuring response inhibition (Verbruggen et al., 2019) and is available under a GNU licence on GitHub (https://github.com/fredvbrug/STOP-IT). For this task participants were presented with left and right pointing arrows (with a black outline and white fill) and were asked to indicate the direction of the arrows using the left and right arrow keys. On some trials participants were presented with a stop signal (the arrow would turn red) to indicate they must not respond. The stop signal was presented with a variable delay after the arrow first appeared. The delay was adjusted depending on the participants’ responses. Failure to stop responses led to a decrease in the delay while success in stopping responses led to an increase in the delay. The delay adjustments were made to converge on a value which resulted in a 50% successful stop rate; that value was used to estimate the stop-signal-response-time (SSRT). The SSRT represents the time needed for the response generated by the stop signal to reach completion and we interpret large SSRTs to be a reflection of weak response inhibition.

#### Learning task

Participants took part in a custom built “game-like” learning task programmed by the first author using jsPsych. Participants were introduced to the learning task by being told that they are part of a research team that is trying to find what a friendly unidentified life form (FULF) likes to eat. The learning task consisted of 116 trials, 110 acquisition trials and 6 test trials. On each trial participants were presented with cues (either one or two images of foods) followed by FULF’s reaction, an outcome, which was a tummy ache or no tummy ache. The cues were randomly selected (from a selection of 11 images) for each participant while the outcomes were the same for all participants, tummy ache being used on reinforced trials (+ trials) and no tummy ache was used for the non-reinforced trials (− trials). Participants were instructed to respond while the food was present, before seeing the reaction, to predict FULF’s reaction. The instructions also asked participants to try to maximise the number of correct predictions and minimise the number of incorrect predictions. The foods were present for 2 s during which the participants had to make a prediction, next the participants were shown the outcome for 1.5 s, and finally a fixation cross was presented for a further 2 s before the next trial started. In addition, after completing the learning trials, participants were asked to first predict then rate the likelihood of specific food item combinations to cause a tummy ache, in a predictive then evaluative summation test. Images illustrating the task are available at https://osf.io/k2zce/.

##### Design

The design used to train conditioned inhibition is shown under the acquisition phase in Table 1. Also in Table 1, after acquisition, there were two test blocks, each containing three conditioned inhibition summation tests. Trials in each phase were randomly ordered independently for each participant subject to the constraint, in the acquisition phase, that no more than two trials of each type could occur in succession. Thus, there were 11 blocks of 10 trials each containing one of each trial type. During this stage cue I was trained to become a conditioned inhibitor by using a dual demonstration. The dual demonstration, during which a conditioned inhibitor indicates non-reinforcement in compound with two separate excitatory cues, has been shown to facilitate acquisition of conditioned inhibition compared with a single demonstration (Williams, 1995). Accordingly, cues A and B were reinforced when they were presented alone, but not when they were presented in compound with cue I. In addition, cues A and B were reinforced when presented in compound with cue J to highlight the fact that it was not enough for cues A and B to be presented in compound in order for them to be non-reinforced, but they need to be in compound with I, the conditioned inhibitor (Williams, 1995). Finally, cues K, L, and M were presented non-reinforced so that there were equal numbers of reinforced and non-reinforced trials on the single cue trials, as well as on the compound cue trials. After acquisition there were two conditioned inhibition summation test blocks. During the first test block (the predictive response summation test) participants responded using the keys just as they had done in the acquisition phase, the transition to the test was explicitly signalled. In the second test block (the evaluative summation test) participants were asked to rate the likelihood of a tummy ache occurring on a scale from 0 to 100. In each summation test, excitatory cue C was presented in compound with the putative inhibitor I. Suppression of responding to test compound CI was assessed relative to responding to C alone in the last block of the acquisition phase and relative to compounds of C with two “associatively neutral” control stimuli, that is, CN and CK. Cue N was novel, but in previous unpublished studies in this laboratory we have observed strong suppression of responding to control compounds containing novel stimuli that may obscure, through floor effects, differences between CI and CN. CK was therefore used as a second control compound to compare with CI.

**Table 1.** Design of the learning task.

Acquisition	Summation: Predictive response	Summation: Evaluative response
A+	CI−	CI?
B+	CN−	CN?
C+	CK−	CK?
AI−		
BI−		
AJ+		
BJ+		
K−		
L−		
M−		

*Note.* Reinforcement, tummy ache, is denoted as “+” while non-reinforcement, no tummy ache is shown as “−.” In the evaluative summation test, “?” indicates that participants were asked to rate the likelihood of a tummy ache on a scale from 0 to 100 rather than using the training keys. Each trial type was presented 11 times in the acquisition phase and once in each of the two summation tests.

##### Task instructions

Based on the recent research of Lee and Lovibond (2021), to further facilitate the training of conditioned inhibition, a causal component was included in the instructions. Lee and Lovibond (2021) showed that implying a causal relationship between cues (the foods in this case) and outcomes (the tummy states in this case) could lead to more robust conditioned inhibition effects. Therefore, our instructions included the following: “So far the research team suspects that there is at least one food which causes FULF to have a tummy ache. Also there may be another food that suppresses FULF’s tummy ache.”

### Data selection and analysis

Eighteen of the 133 participants were excluded entirely from the analysis for failing to complete some parts of the experiment resulting in them missing scores on one or more measures. For the remaining 115 participants we then applied two sequential exclusion criteria which ensured that participants met critical learning thresholds for assessment of associative inhibition, as measured in (1) FN discrimination performance and then in (2) the conditioned inhibition summation tests. We aimed to study variation in strength of inhibition in the FN discrimination and in summation tests so we needed to select suitable participants independently of their performance in these parts of the experiment. To ensure that performance in the FN discrimination was indicative of strength of associative response inhibition we elected to exclude non-learners from the analysis of FN performance—poor FN discrimination would not indicate weak response inhibition learning in people who were simply failing to learn the task overall due to inattention, failure to understand and/or follow task instructions, or due to cognitive overload. We therefore defined learners on the basis of their responses on trials that were not part of the FN discrimination during the last two blocks of the acquisition phase, that is, the last two C+, AJ+, BJ+, K-, L-, and M- trials. This defined 12 trials and participants responding correctly on 10 or more trials were classed as learners. Participants responding correctly on less than 10 trials were classed as non-learners and excluded from further analysis. This cut-off was chosen using the binomial distribution; with p(success) = .5 defined as guessing, the probability of getting 10 or more successes on 12 trials is less than .05. Application of this criterion excluded 16 participants leaving 99 whose FN data was analysed below. Our second exclusion criterion was then applied to select participants for analysis of conditioned inhibition in the summation tests. Again, since we wanted to study variation in performance in this task to assess strength of conditioned inhibition, we elected to exclude participants who failed to learn the FN discrimination. Learning the FN discrimination is a necessary (but not sufficient) condition for acquiring conditioned inhibition and we did not want to confound failure to learn the FN discrimination with weak conditioned inhibition. We used performance in the last two blocks of the FN discrimination (the last two A+, B+, AI–, and BI– trials) to define our eight trial performance criteria. Participants with 7 or more trials correct on this basis were included in the analysis of the conditioned inhibition summation tests (binomial distribution *p*(success = .5) 7 or more successes on 8 trials *p* < .05). This excluded a further 24 participants leaving 75 participants whose conditioned inhibition data was analysed below. Comparisons between included and excluded participants on the non-associative measures of inhibition were made using *t*-tests and the groups did not differ on these measures.

All data analyses were carried out using R (R Core Team, 2021). The main data analyses used generalised linear mixed models, parametric and non-parametric analyses of variance (ANOVAs), and multiple regressions. Post hoc power analyses for regressions looking for links between associative and non-associative measures of inhibition and assuming medium effect sizes were carried out using G*Power version 3.1 (Faul et al., 2009). Code and data are available at https://osf.io/k2zce/.

For the analysis of the FN discrimination a generalised linear mixed model (lmer4 package version 1.1.27.1) for binary data was computed using FN discrimination and block as dependent variables. The model estimated the parameters using a maximum likelihood criterion and a logit link function. For each participant performance was encoded in a 22-element binary vector with 1 s indicating correct responses on both components of the FN discriminations in a block (e.g., an outcome prediction on an A+ trial and no outcome prediction on an AI– trial would be coded 1 but any other pattern would be coded 0). There were 11 blocks for each of the two FN discriminations (A+/AI– and B+/BI–) hence the 22-element binary vector. The model was computed in two stages. In the first stage, we had discrimination and block as fixed factors (discrimination, two levels: FN A+/AI– vs. FN B+/BI–, coded 0, 1 and block: 0–10) and participant as a random factor, meaning that an individual intercept was computed for every participant. Block was reverse coded (e.g., Block 11, coded 0, Block 1 coded 10). Reverse coding of block allowed interpretation of the intercepts as terminal performance, intercepts reflecting the probability of the participant responding correctly in the FN discriminations at the end of the acquisition phase. For this initial model block was allowed to have both a linear and a quadratic term. This model was used to confirm that the two FN discriminations were not learned at different rates.

For the second stage, since the FN discriminations were not learned at different rates, the model was updated by removing the discrimination factor and allowing random quadratic slopes for participants in the random structure. Only quadratic slopes were included in the model as they reflected the performance of the participants more accurately than the linear ones, furthermore by excluding the linear slopes the intercepts could be interpreted as performance at the end of training. The slopes reflect the rate of acquisition of the FN discrimination. The slopes obtained from this second generalised linear mixed model for each participant were then used as measures of FN discrimination performance and included in a series of multiple regressions as dependent variables with the (standardised) non-associative measures of inhibition (BIS-11, BIS, BAS, DD, SSRT) as independent variables.

For the analysis of the summation tests repeated measures ANOVAs were employed, followed up by pairwise comparisons to examine the differences between the test cues CI, CN, CK, and C. Bonferroni corrections were applied to these pairwise comparisons. Non-parametric tests were used for the binary data from the predictive response summation test (Friedman’s ANOVA followed by Wilcoxon matched pairs) and parametric tests were used for the continuous data from the evaluative summation test (parametric ANOVA and Student’s *t*-tests). These tests aimed to assess the reduction in responding to C on compounding with cues: I—putative conditioned inhibitor, K—neutral familiar control, and N—novel control. It was expected that the condition inhibitor would reduce responding more than the control cues K and N. As previously mentioned these two control cues were included in the summation test because novel cues were seen to dramatically reduce responding to compounds in previous studies, therefore we were uncertain about the suitability of N as a control due to possible floor effects.

Following the overall analysis of the conditioned inhibition tests we assessed individual differences in our participants on the basis of their performance in the summation tests. The purpose was to look for links between measures of non-associative inhibition the amount of inhibition shown in the summation tests. Multiple regressions were used in which summation test performance was regressed on non-associative measures of inhibition. We also revisited the FN discrimination analyses by looking at the regressions of the FN coefficients on non-associative measures of inhibition, inhibition independent variables based on the summation tests, and interactions.

For the analysis of the predictive summation test, participants were classified as inhibitors or non-inhibitors using their responses to CN and CI as follows. Participants were classified as inhibitors if cue I reduced responding to C more than cue N (CN-CI), otherwise they were classed as non-inhibitors (Glautier & Brudan, 2019). Since there was only one predictive summation test participants were effectively classed as inhibitors if they responded to CN but not to CI and as non-inhibitors otherwise. The data from the evaluative summation test were analysed following the same steps with the only difference being that a continuous score of conditioned inhibition was computed for every participant using their reported probabilities of tummy ache/no tummy ache. The process described above was repeated using a classification based on the difference between CK and CI but in what follows, for economy of reporting, full regression results are only presented for the CK classification if significant effects were found.

## Results

### Non-associative measures of inhibition

Descriptive statistics on the non-associative measures of inhibition for the 99 participants who passed all the inclusion criteria are provided in Table 2.

**Table 2.** Descriptive statistics for non-associative inhibition.

	Mean	Standard Deviation	Min	Max
BIS-11	59.65	9.66	40	90
BIS	21.76	3.83	10	28
BAS	38.72	5.84	23	50
*k*	0.06	0.32	0.0001	3.06
SSRT	240.41	52.10	76	404

### Acquisition

**Figure 1.**
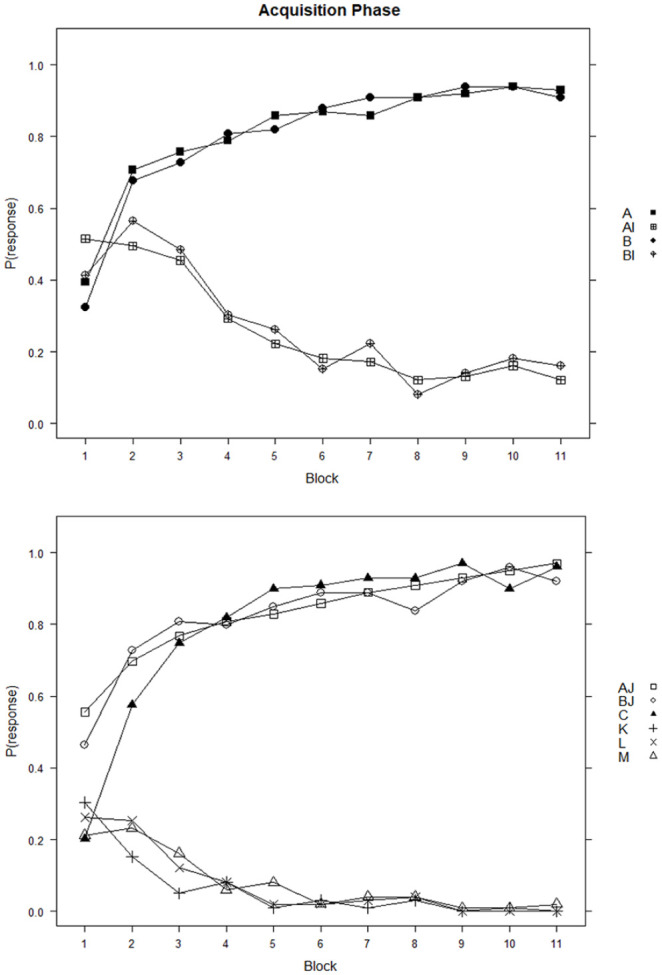
Acquisition phase of the learning task. *Note.* Proportion of trials with an outcome prediction response as a function of block and cue.

The acquisition stage performance of the 99 participants who passed the learning criterion is shown in Figure 1, which indicates that these participants learned to respond more to the reinforced, than to the non-reinforced cues over the course of the acquisition blocks.

#### Feature negative discrimination

An initial generalised linear mixed model with discrimination and block (linear and quadratic) as fixed factors and participants as random factors was used to assess whether the two feature negative discriminations differed. The model revealed that the fixed effects of both the linear and the quadratic terms for block were significant, however the main effect of discrimination was not significant (Table 3). The interactions between discrimination and block (linear), and between discrimination and block (quadratic) were also not significant (Table 3). Accordingly, the initial model showed that overall participants did not perform differently on the A+/AI– and B+/BI– feature negative discriminations during the acquisition phase.

**Table 3.** Feature negative discrimination learning.

Model	Fixed effect	Estimate	*SE*	*z*	*p*
Discrimination × Block	Intercept	0.66	0.13	5.06	<.001
	Discrimination	−0.03	0.11	−0.26	.80
	Block (linear)	−54.45	2.91	−18.70	<.001
	Block (quadratic)	−21.22	2.71	−7.84	<.001
	Discrimination × Block (linear)	−1.29	5.32	−0.24	.81
	Discrimination × Block (quadratic)	−3.77	5.32	−0.71	.48
Block	Intercept	2.24	0.21	10.63	<.001
	Block (quadratic)	−0.04	0.002	−14.77	<.001

*Note.* Initial model with discrimination and block interaction where block had both a linear and quadratic term is presented in the top part of the table, the final model with block quadratic only is presented in the bottom.

As a result, for the final generalised linear mixed model, the discrimination factor and the linear slope were removed, and individual intercepts and quadratic slopes were fitted for each participant (the linear slope was excluded to simplify and to allow for the interpretation of the intercepts produced by the model and due to the fact that learning rates were expected to be quadratic in nature rather than linear). The model revealed that the quadratic effect of block was still significant (Table 3). The individual slopes and intercepts from the model were then used as measures of performance to assess the role of the non-associative measures of inhibition on the FN discrimination learning.

##### Non-associative inhibition

Two multiple regressions were computed using these slopes and intercepts extracted for each participant as the DVs and the non-associative measures of inhibition as IVs. No significant effect of the non-associative inhibition on the FN discrimination learning was found, meaning that the participants’ learning performance was not associated with their performance on the non-associative inhibition tasks/questionnaires (Table 4). Post hoc power was estimated to be 0.84 for these regressions.

**Table 4.** The effect of non-associative inhibition on FN performance.

DV	*R* ^2^	*df*s	*F*	*p*
FN Intercept	.05	5, 93	0.90	.49
	Non-associative inhibition	Unstandardised β	*t*	*p*
	Intercept	0.83	43.22	<.001
	BIS-11	−0.03	−1.40	.16
	BAS	0.006	0.30	.76
	BIS	0.02	0.92	.36
	DD	−0.01	−0.70	.49
	SSRT	0.02	1.18	.24
DV	*R* ^2^	*df*s	F	p
FN Slope Quadratic	.01	5, 93	0.16	.98
	Non-associative Inhibition	Unstandardised β	*t*	*p*
	Intercept	−0.04	−31.85	<.001
	BIS-11	0.001	0.65	.52
	BAS	−0.0003	−0.26	.80
	BIS	−0.001	−0.42	.68
	DD	−0.0001	−0.11	.92
	SSRT	−0.001	−0.45	.65

### Summation test

#### Predictive summation test

**Figure 2.**
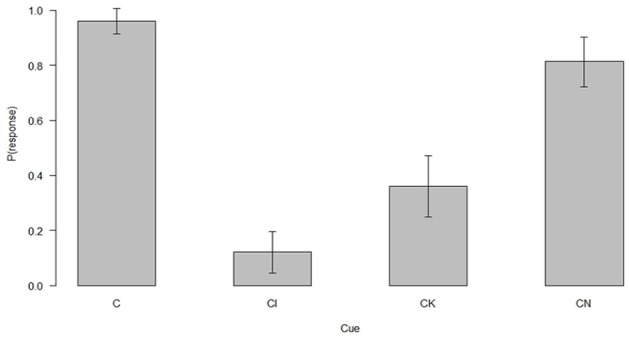
Predictive summation test performance.

The predictive summation test performance of the 75 participants who passed the second exclusion criterion and solved the FN discrimination by the end of the acquisition is presented in Figure 2. Figure 2 shows that responding to CI was markedly suppressed compared with responding to C at the end of the acquisition and compared with control compounds CK and CN.

A Friedman’s ANOVA showed that there were significant differences in responding to the four cues (χ^2^(3) = 124.08, *p* < .001). Follow-up Wilcoxon matched paired tests with a Bonferroni correction showed that CI elicited reduced responding compared with C, CK, and CN (*Z* = –9.94, *p* < .001, *r* = –.92, *Z* = –3.40, *p* = .003, *r* = –.39, and *Z* = –6.83, *p* < .001, *r* = –.79, respectively). Similarly, responses to cues CK and CN were significantly reduced compared with cue C (*Z* = –6.56, *p* < .001, *r* = –.76 and *Z* = –2.67, *p* = .03, *r* = –.31, respectively). Finally, responses to the two control test cues were also significantly different, participants responded less to CK than to CN (*Z* = –5.67, *p* < .001, *r* = –.65).

##### Non-associative inhibition

Participants were classified as inhibitors and non-inhibitors based on their responses to the predictive summation test as described above. There were 55 inhibitors and 20 non-inhibitors in the classification based on CN and 23 inhibitors and 52 non-inhibitors for the classification based on CK. A logistic multiple regression was used to assess whether the non-associative inhibition scores differed for participants classified as inhibitors or non-inhibitors. None of the effects were significant for the CN classification (Table 5) nor for the CK classification. Post hoc power was estimated to be 0.88 for this regression.

**Table 5.** Effects of non-associative inhibition on the predictive summation (CN) test performance.

Model	Cox & Snell *R*^2^	McFadden *R*^2^	*df*s	χ^2^	*p*
	.04	.03	5, 69	2.75	.74
		Non-associative Inhibition	Estimate	Wald Statistic	*p*
		Intercept	1.05	14.56	<.001
		BIS-11	.15	0.28	.60
		BAS	.27	0.94	.33
		BIS	.21	0.67	.41
		DD	−.03	0.01	.91
		SSRT	−.15	0.23	.63

##### Feature negative discrimination and non-associative inhibition revisited

Two multiple regressions with slopes and intercepts as DVs were computed again with an additional factor inhibition group (inhibitor vs. non-inhibitor based on CN) and the interactions between inhibition group and the non-associative measures of inhibition as IVs. The models revealed a significant effect of inhibition grouping on both the intercepts and slopes of the participants. Inhibitors had a better performance on the FN training at the end of training and learnt the FN discrimination faster than the non-inhibitors (Table 6). The regressions were repeated for the classification based on CK but this produced no significant effects. Post hoc power was estimated to be 0.51 for these regressions.

**Table 6.** Effects of non-associative inhibition and inhibition group (predictive summation test CN) on FN performance.

DV	*R* ^2^	*df*s	*F*	*p*
FN Intercept	.13	11, 63	0.88	.57
	Non-associative inhibition	Unstandardised β	*t*	*p*
	Intercept	0.87	40.60	<.001
	BIS-11	−0.03	−0.90	.37
	BAS	−0.005	−0.23	.82
	BIS	−0.01	−0.76	.45
	DD	−0.01	−0.66	.51
	SSRT	0.001	0.02	.98
	Inhibition	0.06	2.37	.02*
	BIS-11 × Inhibition	0.04	1.14	.26
	BAS × Inhibition	−0.01	−0.57	.57
	BIS × Inhibition	0.003	0.11	.91
	DD × Inhibition	0.01	0.44	.66
	SSRT × Inhibition	0.003	0.10	.92
DV	*R* ^2^	*df*s	*F*	*p*
FN Slope Quadratic	.13	11, 63	0.89	.55
	Non-associative Inhibition	Unstandardised β	*t*	*p*
	Intercept	−0.04	−19.25	<.001
	BIS-11	0.001	0.49	.62
	BAS	−0.0004	−0.22	.83
	BIS	0.001	0.69	.49
	DD	0.56	0.57	.57
	SSRT	0.001	0.53	.60
	Inhibition	−0.01	−2.16	.03*
	BIS-11 × Inhibition	−0.002	−0.79	.43
	BAS × Inhibition	0.002	0.82	.42
	BIS × Inhibition	0.0001	0.03	.97
	DD × Inhibition	−0.002	−1.07	.29
	SSRT × Inhibition	−0.001	−0.48	.63

* significance level.

#### Evaluative summation test

**Figure 3.**
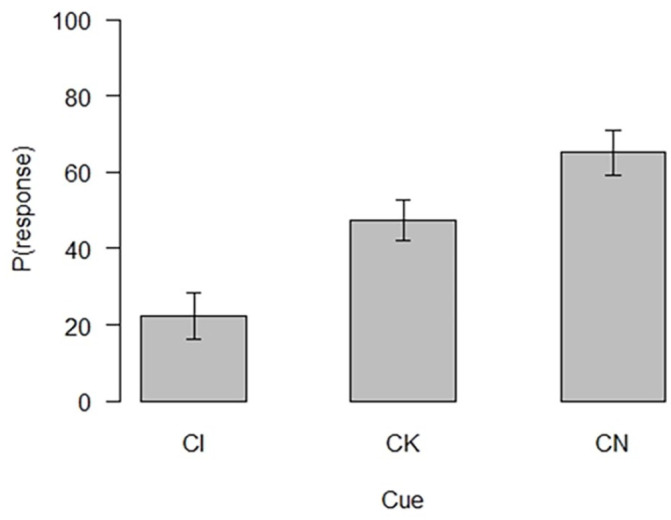
Evaluative summation test performance.

The evaluative summation test performance of the 75 participants who passed the learning criterion is shown in Figure 3.

A repeated measures ANOVA was used to assess the differences between participants’ evaluations of the test cues and C in the last trial of acquisition. The ANOVA revealed a significant effect of cue *F*(2, 148) = 63.19, *p* < .001, *ω*^2^ = .33. All possible comparisons were then computed using paired samples *t*-tests with a Bonferroni correction. The *t*-tests revealed that participants rated the likelihood of CI (*M* = 22.37, *SD* = 26.64) to be reinforced significantly lower than both CK (*M* = 47.53, *SD* = 22.89) *t*(74) = −7.35, *p* < . 001, *d* = −1.70 and CN (*M* = 65.15, *SD* = 25.62) *t*(74) = −9.61, *p* < . 001, *d* = −1.00. These differences confirm the results of the predictive summation test and show that there was an overall effect of conditioned inhibition. The two control compounds, CK and CN, were also rated statistically differently *t*(74) = −5.01, *p* < . 001, *d* = −0.70, CK was rated lower than CN. In summary, all comparisons were significant with compound CI rated the lowest in terms of likelihood of reinforcement, followed by CK and CN in that order (Figure 3).

##### Non-associative inhibition

Inhibition scores were computed for all participants using their evaluative summation test performance as described above. Similarly to the predictive summation test, a linear multiple regression was used to assess whether the non-associative inhibition had an effect on the participants’ inhibition scores. There was a significant effect of BIS on the inhibition scores, participants who have scored high on BIS showed more inhibition on the evaluative summation test (Table 7). None of the other effects were significant. The regression was repeated for the scores based on CK and none of the effects were significant. Post hoc power was estimated to be 0.70 for these regressions.

**Table 7.** Effects of non-associative inhibition on evaluative summation (CN) test performance.

	*R* ^2^	*df*s	*F*	*p*
	.10	5, 69	1.49	.21
	Non-associative Inhibition	Unstandardised β	*t*	*p*
	Intercept	−0.03	−0.23	.82
	BIS-11	−0.12	−1.04	.30
	BAS	−0.12	−1.04	.30
	BIS	0.27	2.28	.03*
	DD	−0.02	−0.13	.90
	SSRT	−0.09	−0.65	.52

* significance level.

##### Feature negative discrimination and non-associative inhibition revisited

Following the methodology set out in the predictive summation test, two multiple regressions with slopes and intercepts as DVs were computed again with inhibition score along with interactions between inhibition score and the non-associative inhibition scores as IVs. None of the effects were significant (Table 8). The regression was repeated with inhibition scores based on CK and this revealed a significant effect of inhibition score on both the learning intercepts and slopes (Table 9). Higher inhibition scores were indicative of larger intercepts and slopes, meaning that participants who showed more inhibition were more likely to have learnt the FN discrimination by the end of the acquisition, and this learning has occurred faster. The effect of inhibition score and SSRT interaction on the intercept was also significant, according to this effect participants who had low SSRT scores (meaning they were fast in stopping their responses) and showed less inhibition performed worse in the FN discrimination at the end of training compared with fast participants who showed more inhibition. On the other hand participants who had high SSRT scores (meaning they were slow in stopping in their responses) showed the same level of performance in the FN discrimination regardless of how much inhibition they showed. None of the other effects were significant. Post hoc power was estimated to be 0.51 for these regressions.

**Table 8.** Effects of non-associative inhibition and inhibition group (evaluative summation test CN) on FN performance.

DV	*R* ^2^	*df*s	*F*	*p*
FN Intercept	.12	11, 63	0.79	.65
	Non-associative inhibition	Unstandardised β	*t*	*p*
	Intercept	0.91	85.52	<.001
	BIS-11	0.01	0.82	.42
	BAS	−0.01	−1.20	.23
	BIS	−0.01	−1.12	.27
	DD	−0.01	−0.94	.35
	SSRT	0.01	1.07	.29
	Inhibition	0.02	1.46	.15
	BIS-11 × Inhibition	0.003	0.31	.76
	BAS × Inhibition	0.01	0.51	.61
	BIS × Inhibition	−0.01	−0.45	.66
	DD × Inhibition	0.01	0.71	.48
	SSRT × Inhibition	−0.03	−1.81	.08
DV	*R* ^2^	*df*s	*F*	*p*
FN Slope Quadratic	.08	11, 63	0.51	.89
	Non-associative inhibition	Unstandardised β	*t*	*p*
	Intercept	−0.04	−40.29	<.001
	BIS-11	−0.001	−1.10	.28
	BAS	0.001	0.69	.50
	BIS	0.001	1.10	.28
	DD	−0.0003	−0.24	.81
	SSRT	−0.001	−0.40	.69
	Inhibition	−0.001	−1.32	.19
	BIS-11 × Inhibition	0.00004	0.04	.97
	BAS × Inhibition	0.0001	0.04	.97
	BIS × Inhibition	0.0002	0.19	.85
	DD × Inhibition	−0.001	−1.11	.27
	SSRT × Inhibition	0.002	0.99	.32

**Table 9.** Effects of non-associative inhibition and inhibition group (evaluative summation test CK) on FN performance.

DV	*R* ^2^	*df*s	*F*	*p*
FN Intercept	.20	11, 63	1.39	.20
	Non-associative inhibition	Unstandardised β	*t*	*p*
	Intercept	0.91	89.75	<.001
	BIS-11	0.01	0.55	.59
	BAS	−0.01	−0.99	.33
	BIS	−0.01	−0.90	.37
	DD	−0.01	−0.81	.42
	SSRT	0.004	0.31	.76
	Inhibition	0.02	2.10	.04*
	BIS-11 × Inhibition	0.01	0.93	.35
	BAS × Inhibition	0.001	0.13	.90
	BIS × Inhibition	−0.001	−0.05	.96
	DD × Inhibition	−0.001	−0.77	.94
	SSRT × Inhibition	−0.03	−2.46	.02*
DV	*R* ^2^	*df*s	*F*	*p*
FN Slope Quadratic	.19	11, 63	1.30	.24
	Non-associative inhibition	Unstandardised β	*t*	*p*
	Intercept	−0.04	−43.62	<.001
	BIS-11	−0.001	−0.77	.44
	BAS	0.0005	0.47	.64
	BIS	0.001	0.91	.36
	DD	−0.0004	−0.31	.76
	SSRT	0.0005	0.42	.68
	Inhibition	−0.02	−2.07	.04*
	BIS-11 × Inhibition	0.0002	0.16	.87
	BAS × Inhibition	−0.001	−1.14	.26
	BIS × Inhibition	−0.001	−0.07	.55
	DD × Inhibition	0.0001	0.07	.95
	SSRT × Inhibition	0.002	1.90	.06

* significance level.

### Additional checks

We elected a priori to carry out our analyses using overall scores on our BIS-11 and BIS/BAS questionnaire measures as we had no strong reasons to anticipate that some subscales on these measures would more strongly linked to associative inhibition than others. However, to check on this exploratory correlations and *t*-tests were carried out the results of which are presented in Table 10. These checks did not reveal any new significant relationships, apart from a significant negative correlation between BAS Reward Responsiveness and the FN intercepts suggesting that higher reward responsiveness was associated with worse performance at the end of training.

**Table 10.** Additional checks of the associative/non-associative inhibition relationship.

	Pred sum CN (t)	Pred sum CK (t)	BIS-11 motor	BIS-11 non-planning	BAS drive	BAS fun seeking	BAS reward responsiveness	BIS	*k*	SSRT	FN intercept	FN slope	Eval sum CN	Eval sum CK
BIS-11 Attentional	0.23	−1.36	.39***	.52***	−.10	.20	.05	.26*	.16	−.02	.01	−.01	.12	.22
BIS-11 Motor	−0.63	−1.36		.50***	.18	.42***	.05	.13	.18	−.05	.04	−.14	−.19	−.11
BIS-11 Non-planning	−0.14	−1.26			−.07	.20	−.11	.12	.05	.08	.02	−.05	−.12	−.10
BAS Drive	−0.20	−0.79				.49***	.44***	−.09	.06	.06	−.10	.12	−.19	−.14
BAS Fun Seeking	−2.05	−2.04					.32***	.01	.08	−.14	.01	−.03	−.10	−.03
BAS Reward Responsiveness	−0.48	−0.50						.40***	.22	.17	−.27*	.14	.05	.02
BIS	−1.11	−0.92							.02	−.09	−.12	.11	.24*	.11
k	−0.002	0.45								.26*	−.07	−.04	−.07	−.11
SSRT	0.55	0.44									.02	.01	−.11	.05
FN Intercept	−1.97	−1.12										−.81***	.15	.24*
FN Slope	2.02	0.83											−.12	−.23*
Eval Sum CN	−3.78*	−3.47*												.63***
Eval Sum CK	−2.75	−5.05*												

*Note*. The first two columns of the table show the *t* values from *t*-tests where all measures of non-associative inhibition were used as dependent variables while the classification into inhibitors/non-inhibitors using both N and K as control stimuli were used as grouping variables. To account for the multiple tests, a Bonferroni correction was applied and the nominal significance level (α = .05) was lowered to .003. The rest of the table shows the correlation matrix of all measures with (uncorrected) *p* < .05, *p* < .01, and *p* < .001 marked by *, **, and ***, respectively.

## Discussion

The current experiment used a feature negative discrimination task to produce associative inhibition along with four measures of non-associative inhibition to assess whether a common underlying inhibitory mechanism exists to link these two domains of inhibition. We determined that participants’ performance in the feature negative discrimination task was not clearly related to any of our non-associative measures of inhibition (BIS/BAS, BIS-11, Delay discounting, and SSRT, Table 4) regardless of whether or not the participants were classed as inhibitors or as non-inhibitors (Table 6) and regardless of whether or not the summation test used to classify participants was based on predictive or evaluative responses (Tables 8 and 9). We did, however, find that inhibitors did tend to perform better in the FN discrimination than the non-inhibitors (Tables 6 and 9) but these effects were not strong nor were they consistent across the two cues used in the evaluative summation tests (Tables 8 and 9). Nevertheless, the fact that effects showed in both the predictive (Table 6) and the evaluative summation tests (Table 9) gives a degree of confidence in this finding. A link between non-associative inhibition and FN performance was seen in Table 9 where there was an interaction between SSRT and inhibition group based on the evaluative summation test using control cue CK using the intercept of the FN discrimination as the dependent variable. However, this was not replicated in the other inhibition group classification (based on CN, Table 8) nor in the inhibition group classification from the predictive summation test (Table 6). With respect to links between the summation measures of associative inhibition and non-associative inhibition measures the only significant result was on the relationship between BIS and performance in the evaluative summation test using control compound CN (Table 7). However this was not replicated using control cue CK nor with the predictive summation test (Table 5). High BIS scores are indicative of a strong inhibitory system, therefore this positive correlation confirmed the a priori expectation of participants with high scores on BIS to show strong inhibition.

Since we applied learning criteria to select only those participants who learned task prerequisites sufficiently well so that their performance could not be easily explained at the level of chance we are confident that performance in the FN discrimination and in the summation tests was actually indicative of the strength of inhibitory learning rather than reflective of a learning deficit. We therefore conclude that, although associative and non-associative inhibition both involve some form of inhibitory process, they are likely to be independent processes. While the application of our learning criteria gives reassurance on critical aspects of data quality the reduction in sample size leads to a caveat to the effect that we cannot be sure that our “null” result would hold with larger samples. Nevertheless, we did carry out some checks which reassure us that if any correlations between associative and non-associative measures do exist then they are unlikely to be large effects. To support this, we carried out post hoc power analyses which showed that our primary tests looking at effects of non-associative inhibition on FN and predictive summation performance had power > 0.8 (Tables 4 and 5). The power of our other tests was lower but > 0.5 in all cases. We also checked to determine whether or not using total questionnaire scores may have masked correlations involving sub-scales (Table 10) but found no evidence that this was the case. A failure to detect correlation effects could also arise if a restricted range of one or more of the variables was sampled. A brief comparison of our self-report mean scores and standard deviations with other published data suggests our sampling was adequate to capture the broad range of values typically encountered (c.f. Table 2); typical BIS-11 total scores are around 62 (*SD* 10) (e.g., Stanford et al., 2009), and for BIS and BAS scores are around 21, 40 (*SD* 3.5, 5) respectively (e.g., Jorm et al., 1998). For behavioural tasks mean values may depend on the precise configuration of the task (e.g., for SSRT the proportion of stop trials can vary and for delay discounting the value of the rewards can vary). Nevertheless for SSRT, Caswell et al. (2015) report (approx.) mean SSRT of 271 ms (*SD* 73) and Hedge et al. (2018) report an (approx.) mean SSRT of 260 ms (*SD* 41). Finding comparisons for the delay discounting task is more difficult but the *k* values we obtained do not seem unusual or restricted compared with others for example Caswell et al. (2015) reported mean values of approx. 0.025 (*SD* 0.09) and Vuchinich and Simpson (1998) reported approx. *k* values of 0.11 (*SD* 0.24). But note that despite what appears to be adequate sampling we have no evidence on what would happen if different participant populations were tested which might, for example, have much higher scores on BIS-11 than undergraduates as was found in Buckfield et al. (2021) where dependent drinkers had mean total BIS-11 score of approx. 75 (*SD* 12). In Buckfield et el., the dependent drinkers did not differ from controls on acquisition of context inhibition during extinction but they did show slower extinction, failing to inhibit responses to a cue that was no longer reinforced.

Various models of inhibition/impulsivity can be found in the literature, however associative inhibition is not often considered in attempting to map these concepts. For example, Caswell et al. (2015) assessed the link between 10 behavioural and one self-reported measure of impulsivity and have proposed a four-factor model of impulsivity. The factors were: motor-impulsivity (action cancellation), reflection-impulsivity, action restraint, temporal-impulsivity. Caswell et al. (2015) aimed to show the multidimensionality of impulsivity, and the four factors proposed along with the fact that four of the measures used did not load on any of the factors supported this view. Based on our current results we suggest that if associative inhibition had been included in this study (and others) then this factorial structure would be altered perhaps with the emergence of an additional “associative inhibition” factor.

Bari and Robbins (2013) proposed a structure for the concept of inhibition which partitions inhibition into cognitive and behavioural inhibition based on the growing number of studies showing a lack of correlation between self-reported and behavioural measures of inhibition (Broos et al., 2012; Enticott et al., 2006; Reynolds et al., 2006). Behavioural inhibition was further subdivided into response inhibition, deferred gratification, and reversal learning. Associative inhibition was included in this proposed underlying structure of inhibition in the form of reversal learning which assesses a participant’s cognitive flexibility and ability to adapt to changes. Although the fact that inhibition/impulsivity are multifaceted concepts is generally accepted, a clear classification of the underlying structure has not been agreed upon. We argue that associative inhibition should be considered as a facet of inhibition in future development of inhibition/impulsivity models.

In isolation associative and non-associative inhibition have been historically shown to have important clinical implications being associated with disorders such as attention-deficit/hyperactivity disorder, substance abuse, and schizophrenia (Bauer, 2001; Enticott et al., 2008; Fillmore & Rush, 2002, 2006; Hoptman et al., 2002; Porter et al., 2011; Schachar et al., 1993). However the two types of inhibition have been rarely used together, one of the few examples is the study by He et al. (2011) that assessed the relationship between conditioned inhibition and personality disorder using a sample of participants with a history of violent offences and a control group from the general population. Although not explicitly measured, impulsivity was assumed to be high in the group of participants with personality disorders and a history of violent offences. The results showed that this group also performed worse in the conditioned inhibition task, showing an impaired ability to develop conditioned inhibition. The results of He et al. (2011) along with our evidence above suggesting that associative and non-associative inhibition are independent processes lead us to conclude that further investigations are warranted (1) to further dissect the nature of the inhibitory deficits underpinning “impulsivity” and (2) to assess the importance of associative inhibition, alongside non-associative inhibition, in psychopathology (e.g., in addiction and personality disorders).

In the foregoing we occasionally used the terms impulsivity and inhibition almost inter-changeably because, even though there have been numerous attempts to define impulsivity without a generally satisfactory solution (e.g., Evenden, 1999; Strickland & Johnson, 2021), it is clear that inhibition must form a substantial part of any general impulsivity definition. Strickland and Johnson rejected the idea that impulsivity has any use as an “umbrella term” (p. 337) and the current work indicates that even focussing down on “inhibition” we still have a very high level and rather abstract concept. This will come as no news to previous investigators (e.g., Bari & Robbins, 2013) but the addition of associative measures of inhibition to the technical and conceptual armoury offers an opportunity to advance the field.

## References

[bibr1-17470218241310859] Bari A. Robbins T. W. (2013). Inhibition and impulsivity: Behavioral and neural basis of response control. Progress in Neurobiology, 108, 44–79. 10.1016/j.pneurobio.2013.06.005 23856628

[bibr2-17470218241310859] Bauer L. O. (2001). Antisocial personality disorder and cocaine dependence: Their effects on behavioral and electroencephalographic measures of time estimation. Drug and Alcohol Dependence, 63(1), 87–95. 10.1016/S0376-8716(00)00195-2 11297834

[bibr3-17470218241310859] Bonardi C. Robinson J. Jennings D. (2017). Can existing associative principles explain occasion setting? Some old ideas and some new data. Behavioural Processes, 137, 5–18. 10.1016/j.beproc.2016.07.007 27425659

[bibr4-17470218241310859] Bouton M. E. (1994). Conditioning, remembering, and forgetting. Journal of Experimental Psychology: Animal Behavior Processes, 20(3), 219–231. 10.1037//0097-7403.20.3.219

[bibr5-17470218241310859] Bouton M. E. (1997). Signals for whether versus when an event will occur. In Bouton M. E. Fanselow M. S. (Eds.), Learning, motivation, and cognition: The functional behaviorism of Robert C. Bolles (pp. 385–409). American Psychological Association. 10.1037/10223-019

[bibr6-17470218241310859] Broos N. Schmaal L. Wiskerke J. Kostelijk L. Lam T. Stoop N. Goudriaan A. E. (2012). The relationship between impulsive choice and impulsive action: A cross-species translational study. PLOS ONE, 7(5), 1–9. 10.1371/journal.pone.0036781 PMC3344935 22574225

[bibr7-17470218241310859] Buckfield C. Sinclair J. M. A. Glautier S. (2021). Slow associative learning in alcohol dependence and the alcohol cue exposure treatment paradox. Addiction, 116, 759–768. 10.1111/add.15210 32725645

[bibr8-17470218241310859] Carver C. S. White T. L. (1994). Behavioral inhibition, behavioral activation, and affective responses to impending reward and punishment: The BIS/BAS scales. Journal of Personality and Social Psychology, 67(2), 319–333. 10.1037/0022-3514.67.2.319

[bibr9-17470218241310859] Caswell A. J. Bond R. Duka T. Morgan M. J. (2015). Further evidence of the heterogeneous nature of impulsivity. Personality and Individual Differences, 76, 68–74. 10.1016/J.PAID.2014.11.059 25844002 PMC4316178

[bibr10-17470218241310859] Enticott P. G. Ogloff J. R. P. Bradshaw J. L. (2006). Associations between laboratory measures of executive inhibitory control and self-reported impulsivity. Personality and Individual Differences, 41(2), 285–294. 10.1016/j.paid.2006.01.011

[bibr11-17470218241310859] Enticott P. G. Ogloff J. R. P. Bradshaw J. L. (2008). Response inhibition and impulsivity in schizophrenia. Psychiatry Research, 157(1–3), 251–254. 10.1016/j.psychres.2007.04.007 17916385

[bibr12-17470218241310859] Evenden J. L. (1999). Varieties of impulsivity. Psychopharm-acology, 146, 348–361. 10.1007/pl00005481 10550486

[bibr13-17470218241310859] Faul F. Erdfelder E. Buchner A. Lang A. G. (2009). Statistical power analyses using G*Power 3.1: Tests for correlation and regression analyses. Behavior Research Methods, 41, 1149–1160. 10.3758/BRM.41.4.1149 19897823

[bibr14-17470218241310859] Fillmore M. T. Rush C. R. (2002). Impaired inhibitory control of behavior in chronic cocaine users. Drug and Alcohol Dependence, 66(3), 265–273. 10.1016/S0376-8716(01)00206-X 12062461

[bibr15-17470218241310859] Fillmore M. T. Rush C. R. (2006). Polydrug abusers display impaired discrimination-reversal learning in a model of behavioural control Sensitivity to the disinhibiting effect of alcohol: The role of trait impulsivity and sex differences View project PDMP by provider View project. Journal of Psychopharmacology, 20, 24–32. 10.1177/0269881105057000 16174667

[bibr16-17470218241310859] Glautier S. Brudan O. (2019). Stable individual differences in occasion setting. Experimental Psychology, 66(4), 281–295. 10.1027/1618-3169/a000453 31530248

[bibr17-17470218241310859] Gray J. A. (1982). The neuropsychology of anxiety: An enquiry into the functions of septo-hippocampal theories. Behavioral and Brain Sciences, 5(3), 492–493. 10.1017/S0140525X00013170

[bibr18-17470218241310859] Gray J. A. (1987). The psychology of fear and stress. Cambridge University Press.

[bibr19-17470218241310859] He Z. Cassaday H. J. Bonardi C. Bibby P. A. (2013). Do personality traits predict individual differences in excitatory and inhibitory learning? Frontiers in Psychology, 4, Article 245. 10.3389/FPSYG.2013.00245/BIBTEX PMC3647220 23658551

[bibr20-17470218241310859] He Z. Cassaday H. J. Howard R. C. Khalifa N. Bonardi C. (2011). Impaired Pavlovian conditioned inhibition in offenders with personality disorders. Quarterly Journal of Experimental Psychology, 64(12), 2334–2351. 10.1080/17470218.2011.616933 22081887

[bibr21-17470218241310859] Hedge C. Powell G. Sumner P. (2018). The reliability paradox: Why robust cognitive tasks do not produce reliable individual differences. Behavior Research Methods, 50(3), 1166–1186. 10.3758/s13428-017-0935-1 28726177 PMC5990556

[bibr22-17470218241310859] Holland P. C. (1992). Occasion setting in Pavlovian conditioning. Psychology of Learning and Motivation—Advances in Research and Theory, 28(C), 69–125. 10.1016/S0079-7421(08)60488-0

[bibr23-17470218241310859] Hoptman M. J. Volavka J. Johnson G. Weiss E. Bilder R. M. Lim K. O. (2002). Frontal white matter microstructure, aggression, and impulsivity in men with schizophrenia: A preliminary study. Biological Psychiatry, 52(1), 9–14. 10.1016/S0006-3223(02)01311-2 12079725

[bibr24-17470218241310859] Jorm A. F. Christensen H. Henderson A. S. Jacomb P. A. Körten A. E. Rodgers B. (1998). Using the BIS/BAS scales to measure behavioural inhibition and behavioural activation: Factor structure, validity and norms in a large community sample. Personality and Individual Differences, 26(1), 49–58. 10.1016/S0191-8869(98)00143-3

[bibr25-17470218241310859] Lee J. C. Lovibond P. F. (2021). Individual differences in causal structures inferred during feature negative learning. Quarterly Journal of Experimental Psychology, 74(1), 150–165. 10.1177/1747021820959286 32988286

[bibr26-17470218241310859] Mazur J. E. (1987). An adjusting procedure for studying delayed reinforcement. In Commons M. L. Mazur J. E. Nevin J. A. Rachlin H. (Eds.), Quantitative analyses of behavior: Vol. 5. The effect of delay and of intervening events on reinforcement value (pp. 55–73). Erlbaum.

[bibr27-17470218241310859] Migo E. M. Corbett K. Graham J. Smith S. Tate S. Moran P. M. Cassaday H. J. (2006). A novel test of conditioned inhibition correlates with personality measures of schizotypy and reward sensitivity. Behavioural Brain Research, 168(2), 299–306. 10.1016/j.bbr.2005.11.021 16386317

[bibr28-17470218241310859] Nelson J. B. (2002). Context specificity of excitation and inhibition in ambiguous stimuli. Learning and Motivation, 33(2), 284–310. 10.1006/lmot.2001.1112

[bibr29-17470218241310859] Patterson C. M. Newman J. P. (1993). Reflectivity and learning from aversive events: Toward a psychological mechanism for the syndromes of disinhibition. Psychological Review, 100(4), 716–736. 10.1037/0033-295x.100.4.716 8255955

[bibr30-17470218241310859] Patton J. H. Stanford M. S. Barratt E. S. (1995). Factor structure of the Barratt impulsiveness scale. Journal of Clinical Psychology, 51(6), 768–774. https://doi.org/10.1002/1097-4679(199511)51:6<768::AID-JCLP2270510607>3.0.CO;2-1 8778124 10.1002/1097-4679(199511)51:6<768::aid-jclp2270510607>3.0.co;2-1

[bibr31-17470218241310859] Porter J. N. Olsen A. S. Gurnsey K. Dugan B. P. Jedema H. P. Bradberry C. W. (2011). Chronic cocaine self-administration in rhesus monkeys: Impact on associative learning, cognitive control, and working memory. Journal of Neuroscience, 31(13), 4926–4934. 10.1523/JNEUROSCI.5426-10.2011 21451031 PMC3099439

[bibr32-17470218241310859] R Core Team (2021). R: A language and environment for statistical computing. R Foundation for Statistical Computing, Vienna, Austria. URL https://www.R-project.org/

[bibr33-17470218241310859] Rescorla R. A. (1987). Facilitation and inhibition. Journal of Experimental Psychology: Animal Behavior Processes, 13(3), 250–259. 10.1037/0097-7403.13.3.250

[bibr34-17470218241310859] Reynolds B. Ortengren A. Richards J. B. de Wit H. (2006). Dimensions of impulsive behavior: Personality and behavioral measures. Personality and Individual Differences, 40(2), 305–315. 10.1016/j.paid.2005.03.024

[bibr35-17470218241310859] Schachar R. J. Tannock R. Logan G. (1993). Inhibitory control, impulsiveness, and attention deficit hyperactivity disorder. Clinical Psychology Review, 13, 721–739. 10.1007/BF01447206 7560554

[bibr36-17470218241310859] Sosa R. (2022). Conditioned inhibition, inhibitory learning, response inhibition, and inhibitory control: Outlining a conceptual clarification. Psychological Review, 131(1), 138–173. 10.1037/rev0000405 36548060

[bibr37-17470218241310859] Sosa R. dos Santos C. V. (2019). Conditioned inhibition and its relationship to impulsivity: Empirical and theoretical considerations. Psychological Record, 69, 315–332. 10.1007/s40732-018-0325-9

[bibr38-17470218241310859] Stanford M. S. Mathias C. W. Dougherty D. M. Lake S. L. Anderson N. E. Patton J. H. (2009). Fifty years of the Barratt Impulsiveness Scale: An update and review. Personality and Individual Differences, 47(5), 385–395. 10.1016/J.PAID.2009.04.008

[bibr39-17470218241310859] Strickland J. C. Johnson M. W. (2021). Rejecting impulsivity as a psychological construct: A theoretical, empirical, and sociocultural argument. Psychological Review, 128(2), 336–361. 10.1037/rev0000263 32969672 PMC8610097

[bibr40-17470218241310859] Swartzentruber D. (1995). Modulatory mechanisms in Pavlovian conditioning. Animal Learning & Behavior, 23(2), 123–143.

[bibr41-17470218241310859] Verbruggen F. Aron A. R. Band G. P. H. Beste C. Bissett P. G. Brockett A. T. Boehler C. N. (2019). A consensus guide to capturing the ability to inhibit actions and impulsive behaviors in the stop-signal task. eLife, 8, Article e46323. 10.7554/ELIFE.46323 PMC6533084 31033438

[bibr42-17470218241310859] Verbruggen F. Logan G. D. (2008). Response inhibition in the stop-signal paradigm. Trends in Cognitive Sciences, 12(11), 418–424. 10.1016/j.tics.2008.07.005 18799345 PMC2709177

[bibr43-17470218241310859] Vuchinich R. E. Simpson C. A. (1998). Hyperbolic temporal discounting in social drinkers and problem drinkers. Experimental and Clinical Psychopharmacology, 6(3), 292–305. 10.1037//1064-1297.6.3.292 9725113

[bibr44-17470218241310859] Williams D. A. (1995). Forms of inhibition in animal and human learning. Journal of Experimental Psychology: Animal Behavior Processes, 21(2), 129–142. 10.1037/0097-7403.21.2.129 7738496

